# Expression optimization, purification, and functional characterization of cholesterol oxidase from *Chromobacterium* sp. DS1

**DOI:** 10.1371/journal.pone.0212217

**Published:** 2019-02-13

**Authors:** Aliakbar Fazaeli, Abolfazl Golestani, Mostafa Lakzaei, Samaneh Sadat Rasi Varaei, Mahdi Aminian

**Affiliations:** 1 Department of Clinical Biochemistry, School of Medicine, Ardabil University of Medical Sciences, Ardabil, Iran; 2 Department of Biochemistry, School of Medicine, Tehran University of Medical Sciences, Ardabil, Iran; 3 Recombinant Vaccine Research Center, Tehran University of Medical Sciences, Tehran, Iran; Universidade Nova de Lisboa, PORTUGAL

## Abstract

Cholesterol oxidase is a bifunctional bacterial flavoenzyme which catalyzes oxidation and isomerization of cholesterol. This valuable enzyme has attracted a great deal of attention because of its wide application in the clinical laboratory, synthesis of steroid derived drugs, food industries, and its potentially insecticidal activity. Therefore, development of an efficient protocol for overproduction of cholesterol oxidase could be valuable and beneficial in this regard. The present study examined the role of various parameters (host strain, culture media, induction time, isopropyl ß-D-1-thiogalactopyranoside concentration, as well as post-induction incubation time and temperature) on over-expression of cholesterol oxidase from *Chromobacterium* sp. DS1. Applying the optimized protocol, the yield of recombinant cholesterol oxidase significantly increased from 92 U/L to 2115 U/L. Under the optimized conditions, the enzyme was produced on a large-scale, and overexpressed cholesterol oxidase was purified from cell lysate by column nickel affinity chromatography. K_m_ and V_max_ values of the purified enzyme for cholesterol were estimated using Lineweaver-Burk plot. Further, the optimum pH and optimum temperature for the enzyme activity were determined. This study reports a straightforward protocol for cholesterol oxidase production which can be performed in any laboratory.

## Introduction

Cholesterol oxidase (EC 1.1.3.6) is a bacterial flavoenzyme belonging to the oxidoreductase enzyme family and catalyzing the oxidation and isomerization of cholesterol as the first step in the cholesterol catabolism. The enzyme structure includes two domains: the FAD binding domain and the substrate binding domain [1]. The enzyme is classified in two types according to the nature of the bond between FAD cofactor and apoenzyme: Type I, the FAD cofactor non-covalently linked to the protein; and Type II, the cofactor covalently bound to the apoenzyme [2]. Both types of enzymes have found wide applications as useful biotechnological tools.

Cholesterol oxidase (COX) is mainly used in clinical laboratories for determining the cholesterol level both in serum and in other biological samples [3]. Further, COX has become valuable for the pharmaceutical industry because of the ability for bioconversion of 3β-hydroxysteroids [4]. Meanwhile, many attempts have been made to reduce cholesterol content of foods by the COX [5–7]. In addition, many reports have addressed the role of COX in pest control strategies [8, 9].

Cholesterol oxidase producing bacteria are divided into two groups: nonpathogenic and pathogenic bacteria. Nonpathogenic bacteria employ COX as a metabolic tool for obtaining carbon sources from cholesterol decomposition, while pathogenic bacteria tend to utilize the enzyme to infect the host macrophages through oxidation of membrane cholesterol [10]. Thanks to the wide applications of cholesterol oxidase in various fields, many efforts have been made to produce a recombinant form of the enzyme in expression hosts. So far, many *COX* genes from different bacterial sources have been cloned and expressed [11–26].

Cholesterol oxidase from *Chromobacterium* sp. DS1 (ChO) was purified and characterized by Doukyu et al. [27]. The purified enzyme molecular mass was 58 kDa and oxidized cholesterol to hydroperoxy-cholest-4-en-3-one (HCEO) through consuming 2 moles of O_2_. Furthermore, it has been reported that the enzyme is very stable at high temperatures and in the presence of detergents and organic solvents, when compared to the commercially available cholesterol oxidases [27]. These features make ChO an appropriate enzyme both for industrial uses and clinical laboratory practice.

Recombinant ChO production in a large quantity facilitates its biochemical characterization and its use in industrial or clinical processes. To this end, in the current study, we have devised a straightforward and effective approach for maximizing ChO production by optimizing the shake flask culture conditions.

## Materials and methods

### Strains, materials, and culture media

*E*. *coli* host strains *BL*21*(DE*3*)*, *BL*21*(DE*3*)pLysS*, and *Rosetta-gami*2*(DE*3*)* were obtained from Novagen (Madison, WI, USA). Synthesis of plasmid pET24b-*ChO* was ordered to Bio Basic Inc. (ON, Canada). Ni-CAM HC Resin, isopropyl-β-D-thiogalactopyranoside (IPTG), kanamycin and chloramphenicol were purchased from Sigma-Aldrich (MO, USA). All other chemicals were prepared from Merck chemical company (Darmstadt, Germany). The following liquid media were used: Luria–Bertani (LB, 10 g/L peptone, 5 g/L yeast extract, 5 g/L NaCl, Merck), Super Broth (SB, 32 g/L peptone, 20 g/L yeast extract and 5 g/L NaCl, Merck), Terrific Broth (TB, 12 g/L peptone, 24 g/L yeast extract, 8 g/L glycerol, 17 mM KH_2_PO_4_ and 72 mM K_2_HPO_4_, Merck).

### Optimization of recombinant ChO expression

#### Expression of ChO in different *E*. *coli* hosts

Initially, the ability of three different *E*. *coli* strains for producing our desired enzyme was investigated. For this purpose, *Chromobacterium* sp. DS1 cholesterol oxidase gene (GenBank accession number **AB456533.1**) was designed in pET24b(+) plasmid among NdeI-BamHI restriction sites (GenBank accession number **MH892608**) and then chemically transformed into *BL*21*(DE*3*)*, *BL*21*(DE*3*)pLysS*, and *Rosetta-gami*2*(DE*3*)* hosts. The transformed cells were plated in LB agar containing 50 μg/μL of kanamycin and additional 25 μg/μL of chloramphenicol in the cases of *BL*21*(DE*3*)pLysS* and *Rosetta-gami*2*(DE*3*)*. After overnight incubation, a single colony of each strain was grown overnight in 3 mL of LB medium containing the mentioned antibiotics on a rotary shaker (160 rpm) at 37°C. On the next day, 10 mL of LB/antibiotics medium was inoculated by 100 μL of pre-culture medium and incubated under the same conditions. Once the cells reached the mid-exponential phase (OD_600 nm_ ≃ 0.6), induction of protein expression was performed with addition of 0.5 mM IPTG and incubated at 37°C while shaking for 6 h. Next, the cells were harvested by centrifugation at 7000 x *g*, 4°C, and 10 min, and then the cells were resuspended in 0.5 mL of PBS buffer at pH 7.0. The suspended cells were disrupted by sonication for 10 min at a frequency of 20 kHz, amplitude of 75%, and duty cycle of 0.7 s and the lysate was centrifuged for 20 min at 13000 x *g*, 4°C. Enzyme activity assay was conducted based on the method of Allain et al. on the crude extract [27, 28]. The assay mixture contained 100 mM potassium phosphate pH 7.0, 1 mM cholesterol, 21 mM phenol, 1.4 mM 4-aminoantipyrine and 5 U/mL peroxidase. The reaction was started by addition of 100 μL sample to 1 mL assay mixture and the appearance of the red chromophore was monitored continuously at 500 nm and the experimental results were reported as mean of three experiments ± SD. Blanks without enzyme or without cholesterol were routinely run in parallel. One unit of activity was defined as the formation of 1 μmol of hydrogen peroxide (0.5 μmol of quioneimine dye) per min at 25°C.

#### Culture media optimization

To determine the effect of different culture media on the yield of active ChO, three types of media were evaluated (LB, TB, and SB). The overnight culture of selected *Rosetta-gami*2*(DE*3*)* harboring pET24-*ChO* plasmid was made in 3 mL of LB medium. Then, 10 mL of each medium was inoculated with the pre-culture bacteria with the ratio of 1:100. When OD_600 nm_ was reached at 0.6, the cultures were induced with 0.5 mM of IPTG and incubated at 37°C, 160 rpm for 6 h. The cultures were harvested and the pellets were resuspended in 0.5 mL of PBS buffer. After sonication, the cell lysate was centrifuged at 13000 x *g*, 4°C, and 20 min. The total activity of the recombinant enzyme was measured by performing enzyme assay in the crude extract for production determination.

#### Optimum induction time

Four shaking flasks containing 10 mL of TB/Antibiotics medium were inoculated by 100 μL of overnight cultured *Rosetta-gami*2*(DE*3*)*-pET24-*ChO* and incubated at 37°C, 160 rpm. When OD_600 nm_ of the cultures reached 0.3, 0.6, 1.2, and 1.8, induction was made through 0.5 mM IPTG. The induced cultures were incubated for 6 h at 37°C, 160 rpm. After disruption of harvested cells and centrifugation, quantification of active enzyme was performed by enzyme activity assay.

#### Optimum inducer concentration

*Rosetta-gami*2*(DE*3*)* cells containing pET24-*ChO* were grown overnight in LB/Antibiotics medium. Fresh cultures (5 flasks) containing 10 mL TB medium were inoculated (1:100) and incubated at 37°C, 160 rpm. When the OD_600 nm_ of cultures reached 0.6, induction was made individually by IPTG concentrations of 0.05, 0.1, 0.25, 0.5, and 1 mM. After 6 h of incubation at 37°C, 160 rpm, the cells were harvested and disrupted by sonication and then the enzyme activity was measured.

#### Induction temperature and post-induction incubation time

The effect of different incubation temperatures (25°C, 30°C, and 37°C) as well as four post-induction incubation times (6, 8, 16, and 24 h) was evaluated on the ChO production. For this purpose, three flasks containing 20 mL of TB/Antibiotics medium were inoculated by 0.2 mL pre-cultured *Rosetta-gami*2*(DE*3*)* harboring *ChO* gene, where the induction was made at OD_600 nm_ ≃ 0.6 by IPTG at a final concentration of 0.1 mM. After induction, each flask was shaken at 160 rpm and 25°C, 30°C, and 37°C. Appropriate post-induction incubation time was determined through withdrawing 2 mL of culture medium from each flask at different time intervals (6, 8, 16, and 24 h). The collected samples were centrifuged and pellets were resuspended in the buffer, and then the cells were disrupted by sonication. Enzyme activity assay performed for quantification of the expressed recombinant enzyme in the prepared crude extract.

### Large-scale production of recombinant ChO

Large-scale production of ChO was performed under optimized conditions. Pre-culture was made by 5 mL of LB [kanamycin (50μg/mL) and chloramphenicol (25μg/mL)] inoculated by 50 μL of *Rosetta-gami*2*(DE*3*)-*pET24*-ChO* glycerol stock. Subsequently, 500 mL of TB/Antibiotics was inoculated by 5 mL of pre-cultured media. When OD_600 nm_ reached 0.6, IPTG (0.1 mM) was added and induction of *ChO* gene expression was done at 30°C, 160 rpm for 16 h. The harvested bacterial pellet was resuspended in 10 mL of resuspension buffer (PBS, NaCl 0.3 M, Imidazole 5 mM, pH 7) and disrupted by sonication. The cell lysate was centrifuged at 13000 x *g*, 4°C, for 20 min with the supernatant used for ChO purification via affinity chromatography.

### Purification of recombinant ChO

Nickel-chelating affinity chromatography (Ni-CAM HC Resin-Sigma) was used for purification of recombinant ChO. For this purpose, initially the column (2 mL) was equilibrated with 30 mL of equilibration buffer (PBS, NaCl 0.3 M, Imidazole 5 mM; pH 7.0) at 1 mL/min. Then, 10 mL of the enzyme crude extract containing 210 mg protein was loaded onto the column, where the column was washed with equilibrium buffer until the absorbance at 280 nm reached the basal level. To elute proteins, elution buffer (PBS, NaCl 0.3 M, and Imidazole 150 mM; pH 7.0) was used and the released proteins were fractionated. The purity of fractionated samples was evaluated by SDS-PAGE 10%. The pure fractions were pooled together and dialyzed against 50 mM sodium phosphate buffer at 4°C, pH 7.0 for 16 h. To determine the purification yield and specific activity of the recombinant ChO, the protein concentration and enzyme activity of the samples were measured by Bradford [29] and enzyme assay, respectively.

### Kinetic characterization of purified ChO

The recombinant ChO activity was assayed at different temperatures (30°C– 80°C) in order to determine the recombinant enzyme optimum thermal activity. The optimum pH of the recombinant enzyme activity was also determined by the enzyme activity assay at 25°C under various pH (3–11) conditions. The buffer systems were prepared according to Doukyu et al. [27]. The K_m_ and V_max_ values for cholesterol was estimated based on Lineweaver-Burk plots of the data obtained from the assay solution containing 0 to 1 mM of cholesterol substrate.

## Results

### Optimization of recombinant ChO expression

#### Optimal host strain for ChO expression

**Fig 1:**
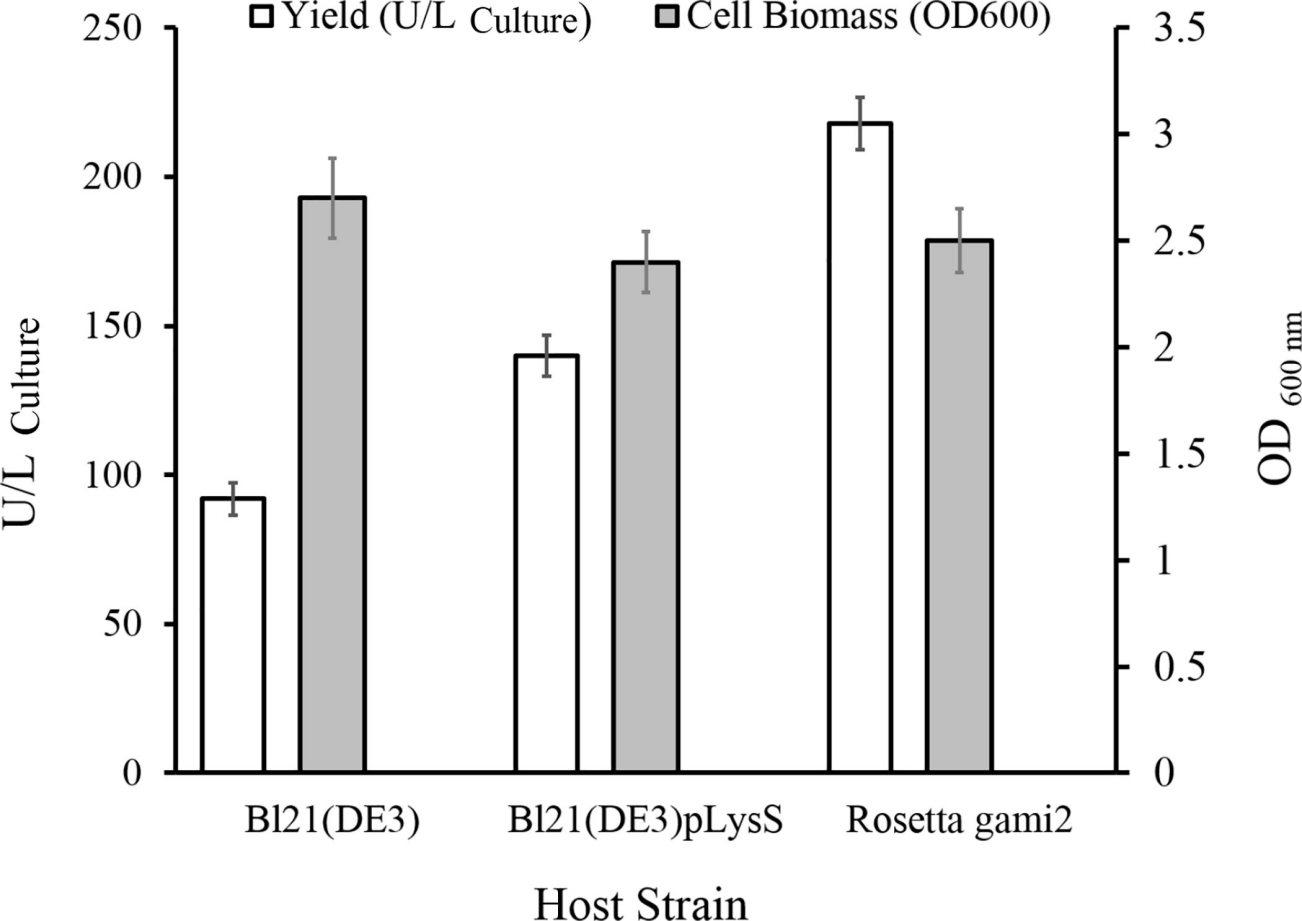
Cholesterol oxidase expression in various host strains. The influence of host strain on the recombinant enzyme yield and bacterial biomass accumulation was evaluated under 6-h cultivation at 37°C.

The ability of *BL*21*(DE*3*)*, *BL*21*(DE*3*)pLysS*, and *Rosetta-gami*2*(DE*3*)* for producing recombinant ChO was evaluated using shake-flask culture. Accordingly, for all of the three cultures, induction were made by 0.5 mM IPTG when OD_600 nm_ reached 0.6 and protein expression continued for 6 h at 37°C, 160 rpm. After sonication, the cell lysates were centrifuged and the supernatants were recovered as crude lysates to determine units of enzyme activity produced per liter of culture medium. The *Rosetta-gami*2*(DE*3*)* cells yielded the highest level of active recombinant ChO with 218 U/L activity (Fig 1). In addition, the total magnitude of active enzyme obtained from *BL*21*(DE*3*)pLysS* was higher compared to the value obtained from *BL*21*(DE*3*)*.

#### Optimal culture medium for ChO expression

**Fig 2:**
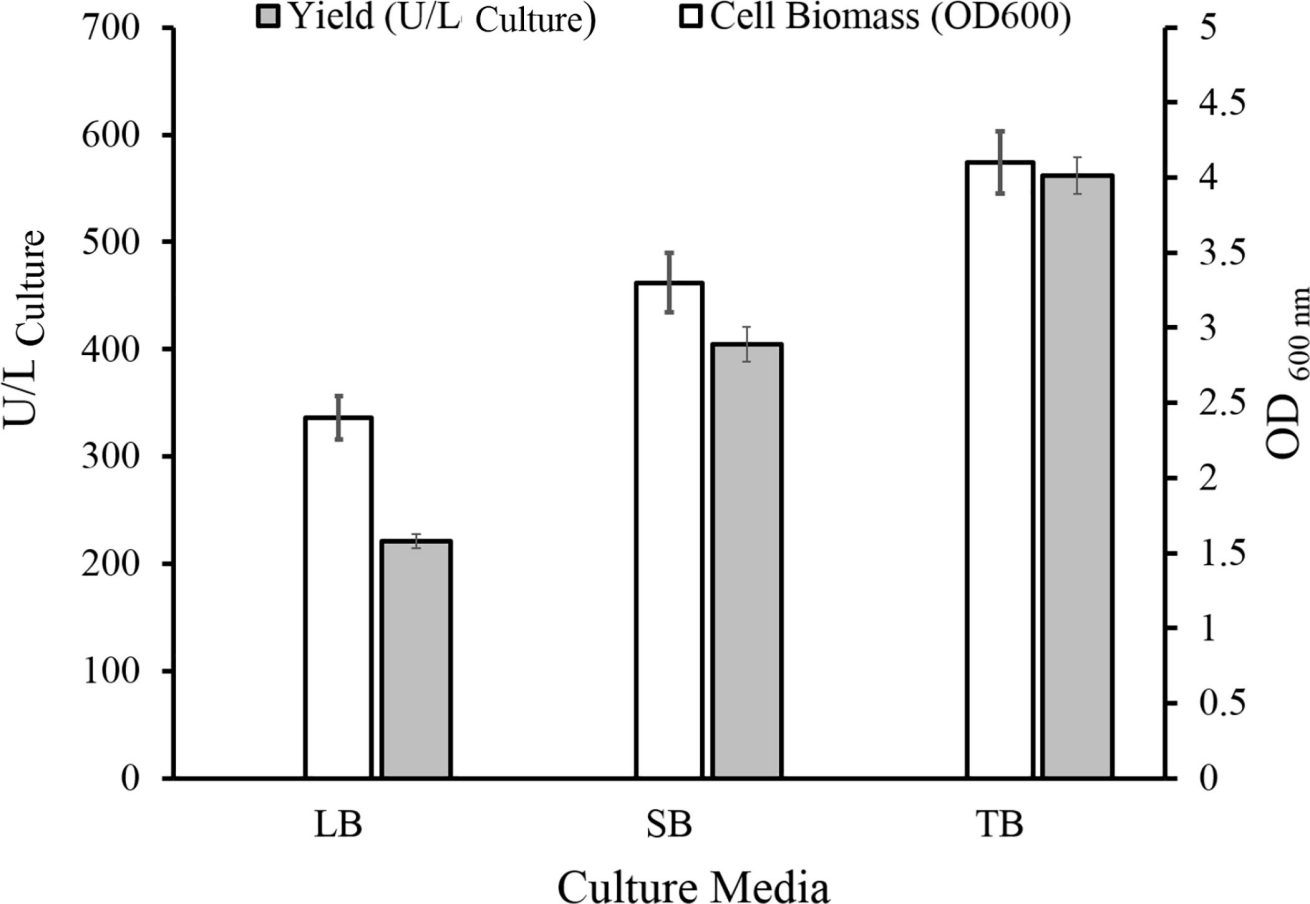
The effect of different types of culture media on cholesterol oxidase production and biomass accumulation. *Rosetta-gami*2*(DE*3*)*-pET24-*ChO* was cultured in LB, SB, and TB media for 6 h post induction incubation time at 37°C.

Three different culture media (LB, TB, and SB) were evaluated to achieve the optimum production of soluble ChO by the *Rosetta-gami*2*(DE*3*)* cells. To compare the effect of different culture media, overnight culture of *Rosetta-gami*2*(DE*3*)* containing pET24-*ChO* was made in LB medium at 37°C. Freshly prepared LB, TB, and SB media were inoculated by pre-culture inoculum (1%) and incubated at 37°C until OD_600 nm_ was reached 0.6. Subsequently, the cultures were induced with 0.5 mM IPTG and were grown for another 6 h at 37°C, 160 rpm. Cell biomass and total enzyme activity obtained by different media types are revealed in Fig 2. The highest cell biomass (OD_600 nm_ = 4.1) was achieved by TB medium. Also, enzyme assay indicated that TB medium supports high production of recombinant ChO in comparison to the other media.

#### Pre-induction growth optimization

**Fig 3:**
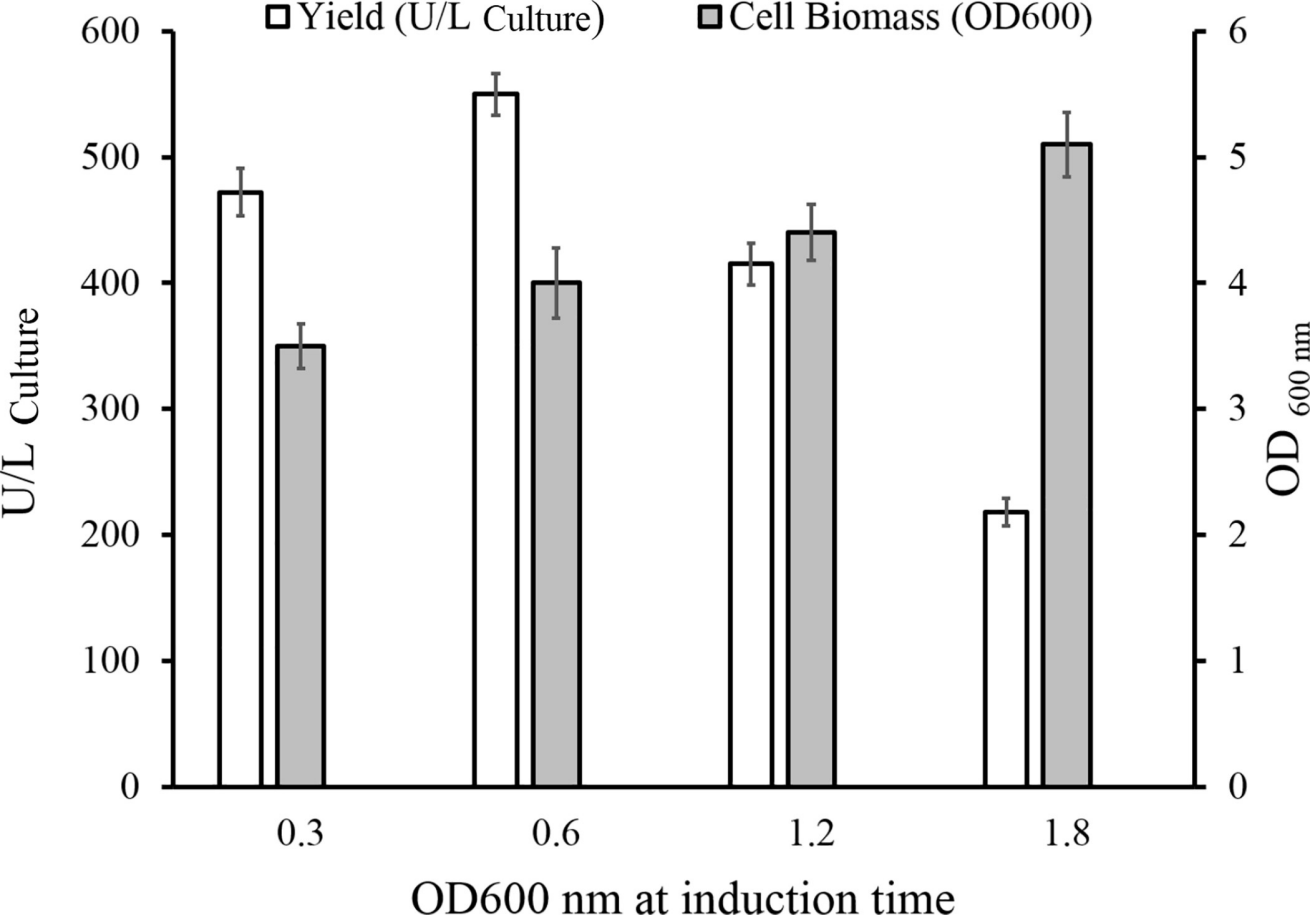
The impact of pre-induction growth on the cholesterol oxidase expression and final growth rate. Protein expression was induced in various bacterial growth phases (OD _600nm_ = 0.3, 0.6, 1.2, and 1.8). Total enzyme activity and final bacterial growth rate were measured after further incubation for 6 h at 37°C.

To determine the best *Rosetta-gami*2*(DE*3*)*-pET24-*ChO* growth phase for induction of ChO production, four shake flasks were examined in parallel, and each culture was induced at different growth phases. ChO production was induced with 0.5 mM IPTG in early exponential (0.3), mid-exponential (0.6), late exponential (1.2), and stationary (1.8) growth phases. The results, represented in Fig 3, indicate that the highest yield of active enzyme (550 U/L) was obtained when induction occurred in the mid-exponential (0.6) growth phase. Furthermore, higher cell biomass (OD_600 nm_ = 5.1) accumulation was observed when induction was made in the stationary (1.8) growth phase.

#### Inducer concentration optimization

**Fig 4:**
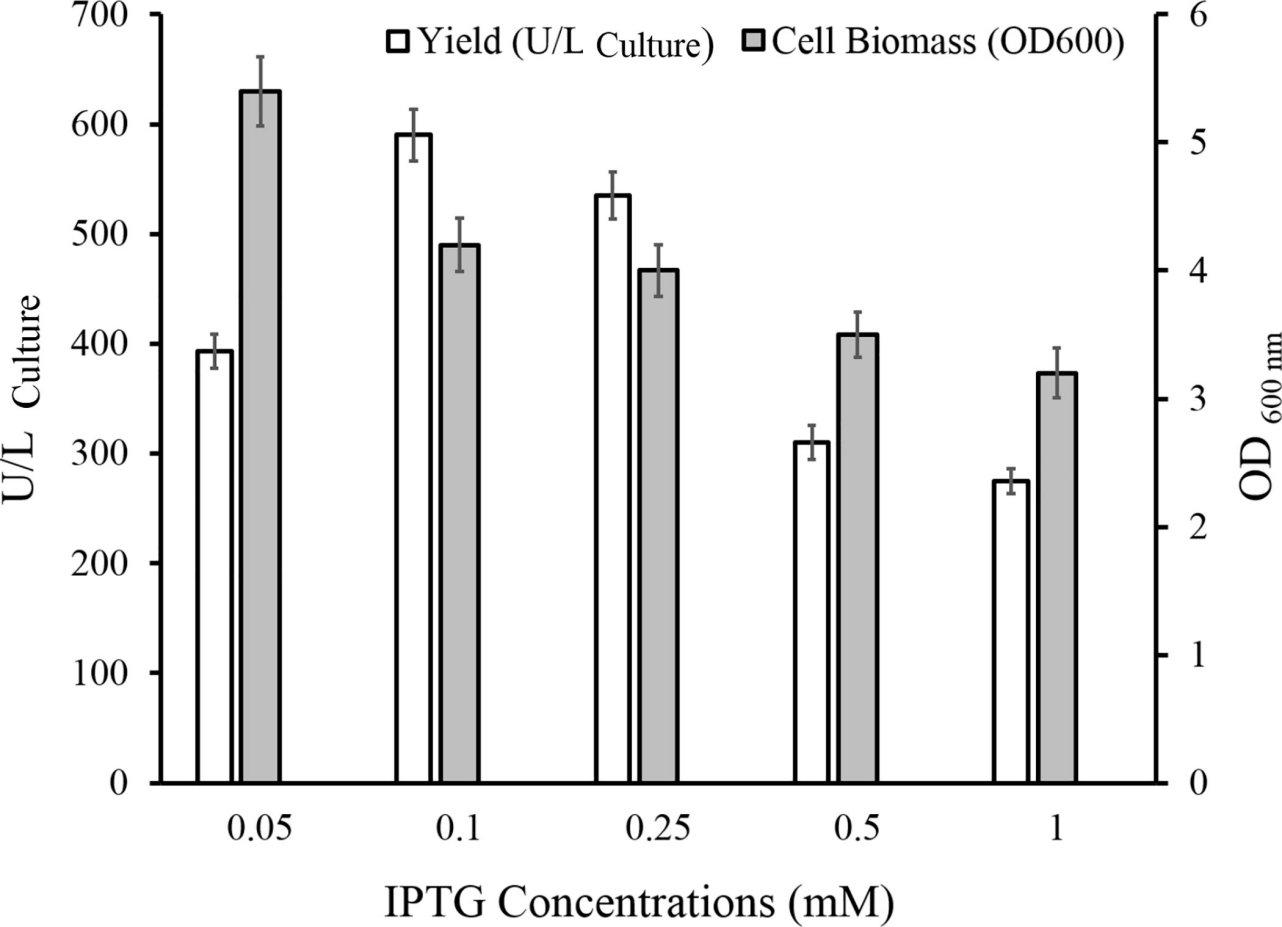
Total cholesterol oxidase activity levels and cell growth at various IPTG concentrations. IPTG concentrations of 0.05, 0.1, 0.25, 0.5, and 1 mM were added to the culture in the mid-exponential growth phase. After 6 h incubation at 37°C, the recombinant enzyme production and cell biomass production were determined.

The influence of inducer concentrations on the expression of ChO was monitored by addition of IPTG to the final concentrations of 0.05, 0.1, 0.25, 0.5, and 1 mM. According to the results, as depicted in Fig 4, induction by 0.1 mM IPTG offered the highest yield of the recombinant enzyme (590 U/L) in comparison to the other IPTG concentrations. Further, cell biomass accumulation decreased along with a gradual growth of IPTG concentration.

#### Optimal induction temperature and post-induction incubation time

**Fig 5:**
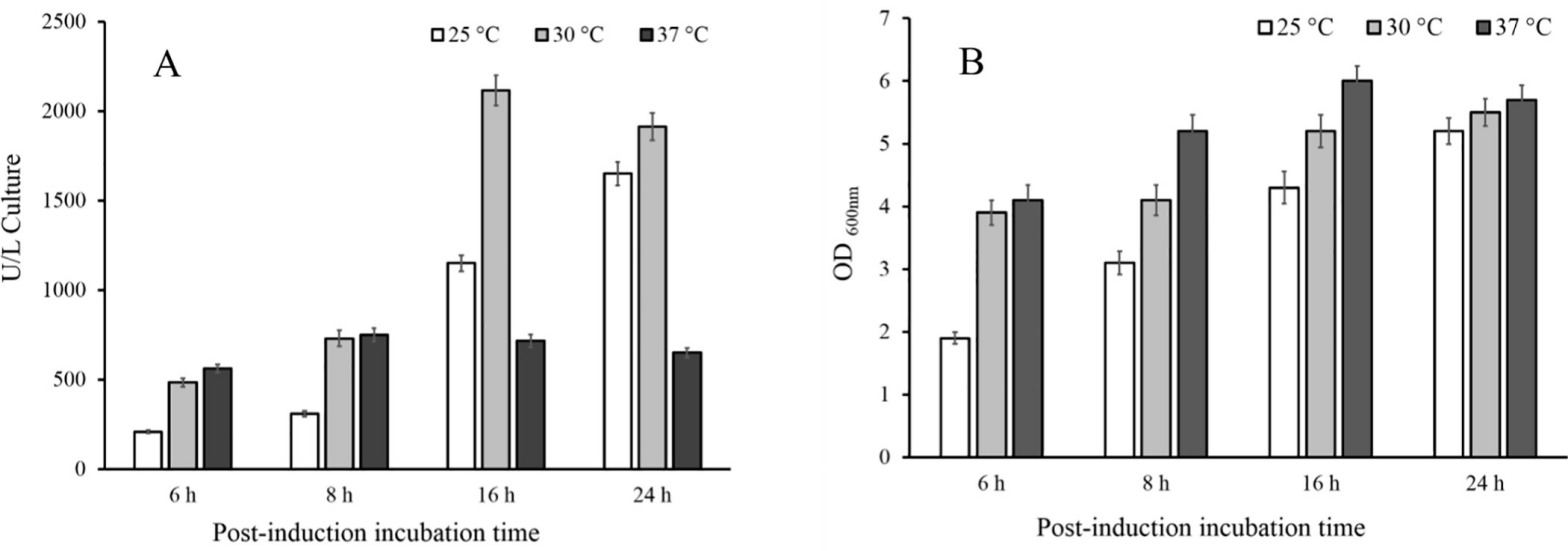
Comparison of cholesterol oxidase production and cell growth at various post-induction incubation times and temperatures. (**A)** Total enzyme activity was measured after various temperatures during the indicated period of time; (**B)** Final biomass production determined after different post-incubation temperatures and times.

Under the best conditions achieved so far [*Rosetta-gami*2*(DE*3*)*, TB medium, induction at OD_600 nm_ = 0.6, and 0.1 mM IPTG], the effect of various induction temperatures as well as post-induction incubation times was investigated on ChO production. To this end, three flasks containing TB medium were cultivated under optimized conditions. After addition of IPTG (0.1 mM), the flasks were incubated at 25°C, 30°C, and 37°C, separately, with 2 mL samples withdrawn from each flask at different time intervals (6, 8, 16, and 24 h). ChO activity assay showed that the yield of recombinant active enzyme markedly increased when the induced culture medium was incubated at 30°C for 16 h. As represented in Fig 5, the total enzyme activity decreased when cultures were incubated under 37°C even for 16 or 24 h.

### Large-scale enzyme production

Putting all the optimal conditions together, we made 500 mL culture of *Rosetta-gami*2*(DE*3*)*-pET24-*ChO* at 30°C for 16 h through induction by 0.1 mM IPTG in the mid-exponential growth phase (OD_600 nm_ ≃ 0.6). After the enzyme crude extract preparation, the total enzyme activity and total protein concentration were measured. As reported in Table 1, the total enzyme activity and total protein content were 1160 U and 380 mg for 500 mL of the culture, respectively.

**Table 1:** Summary of the purification procedure for the recombinant cholesterol oxidase.

Steps	Total activity ^a^ (U)	Total protein (mg)	Specific activity (U/mg)	Purification (fold)	Yield (%)
Crude extract ^b^	972	210	4.6	1	100
Ni-CAM affinity Chromatography	802	56	14.3	3.1	82.5

^a^ Cholesterol oxidation activity was assayed by measuring H_2_O_2_ generation

^b^ Crude extract was obtained from 500 ml of the culture of *Rosetta-gami*2*(DE*3*)*-pET24-*Cho*

### Purification of recombinant ChO

**Fig 6:**
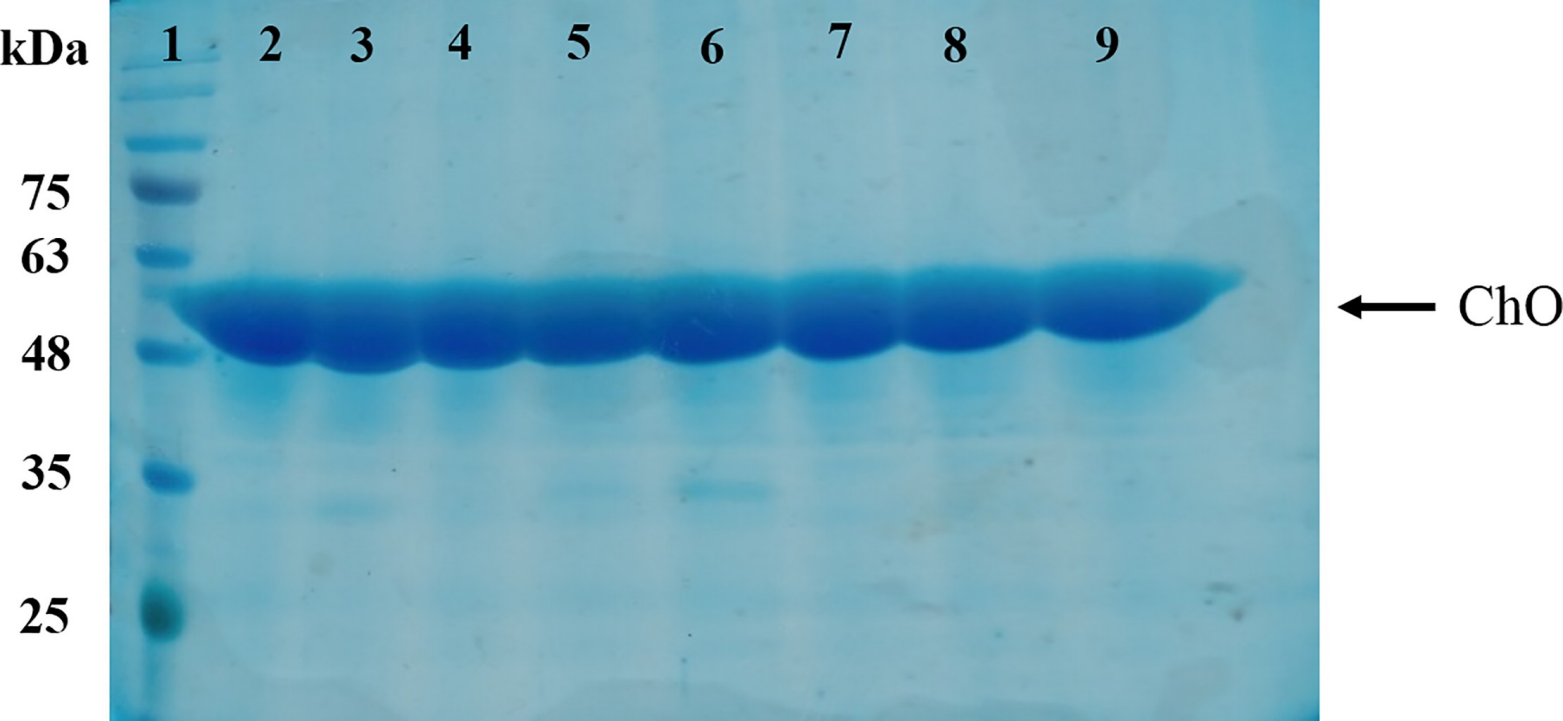
SDS-PAGE analysis of purified recombinant cholesterol oxidase. The quality of the eluted pure recombinant ChO was analyzed using SDS-PAGE 10%. Lane 1: Protein marker; Lanes 2–9: Fractionated samples eluted from Ni-CAM column by 150 mM imidazole.

Nickel column affinity chromatography was used for purification of the recombinant ChO which contained 6×His -Tag in its N-terminal. The result obtained from SDS-PAGE analysis of eluted fractions indicated that pure ChO was mainly eluted by 150 mM imidazole. As displayed in Fig 6, according to lanes 2–9, ChO was highly purified. Fractions containing pure ChO were pooled and dialyzed against 50 mM sodium phosphate buffer at pH 7. Table 1 summarizes the data of purification steps. The overall yield of 82.5% and the overall purification growth of 3.1 times were achieved through Ni-CAM affinity chromatography.

### Properties of the purified cholesterol oxidase

**Fig 7:**
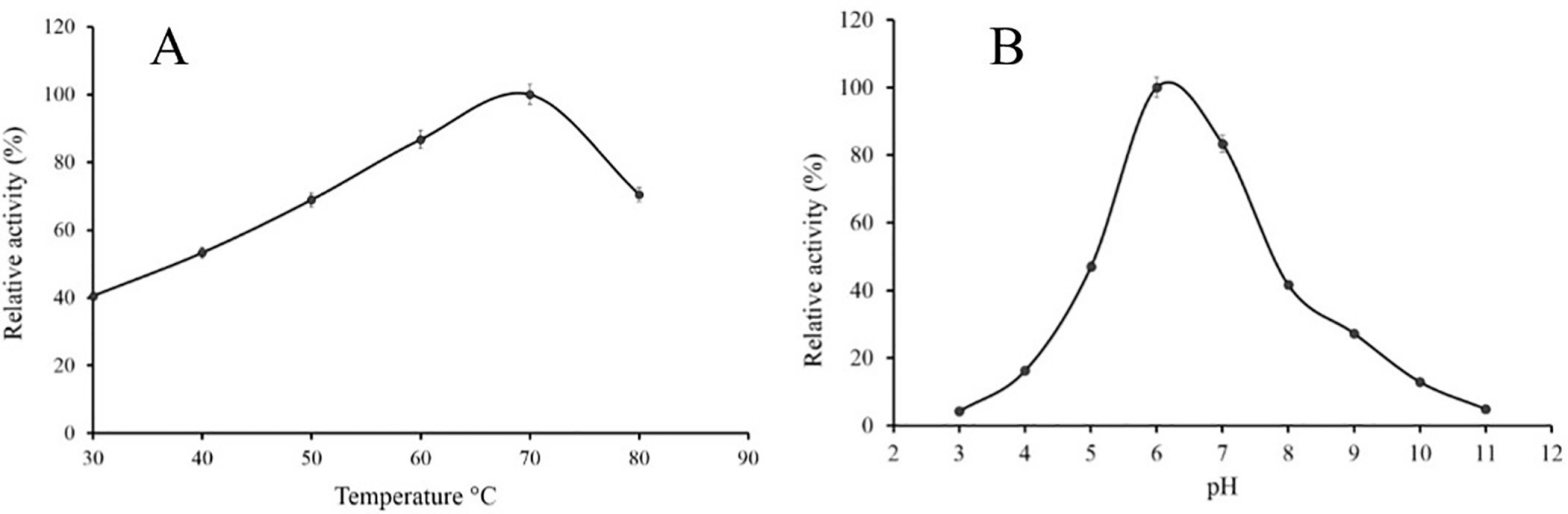
Effect of different pH and temperatures on the activity of the recombinant cholesterol oxidase. **(A)** Effect of temperature: enzyme activity was assayed in potassium phosphate 0.1 mM, pH 7.0 based on the formation of H_2_O_2_ at the indicated temperatures. **(B)** Effect of pH: The residual activity was examined by monitoring H_2_O_2_ generation at 25°C. The buffer systems (0.1 M) utilized were glycine-HCl (pH 3.0), citrate-sodium citrate (pH 4.0), CH_3_COOH-CH_3_COONa (pH 5.0), NaH_2_PO_4_-Na_2_HPO_4_ (pH 6.0), Tris-HCl (pH 7.0–9.0), and Na_2_CO_3_-NaHCO_3_ (pH 10.0–11.0).

**Fig 8:**
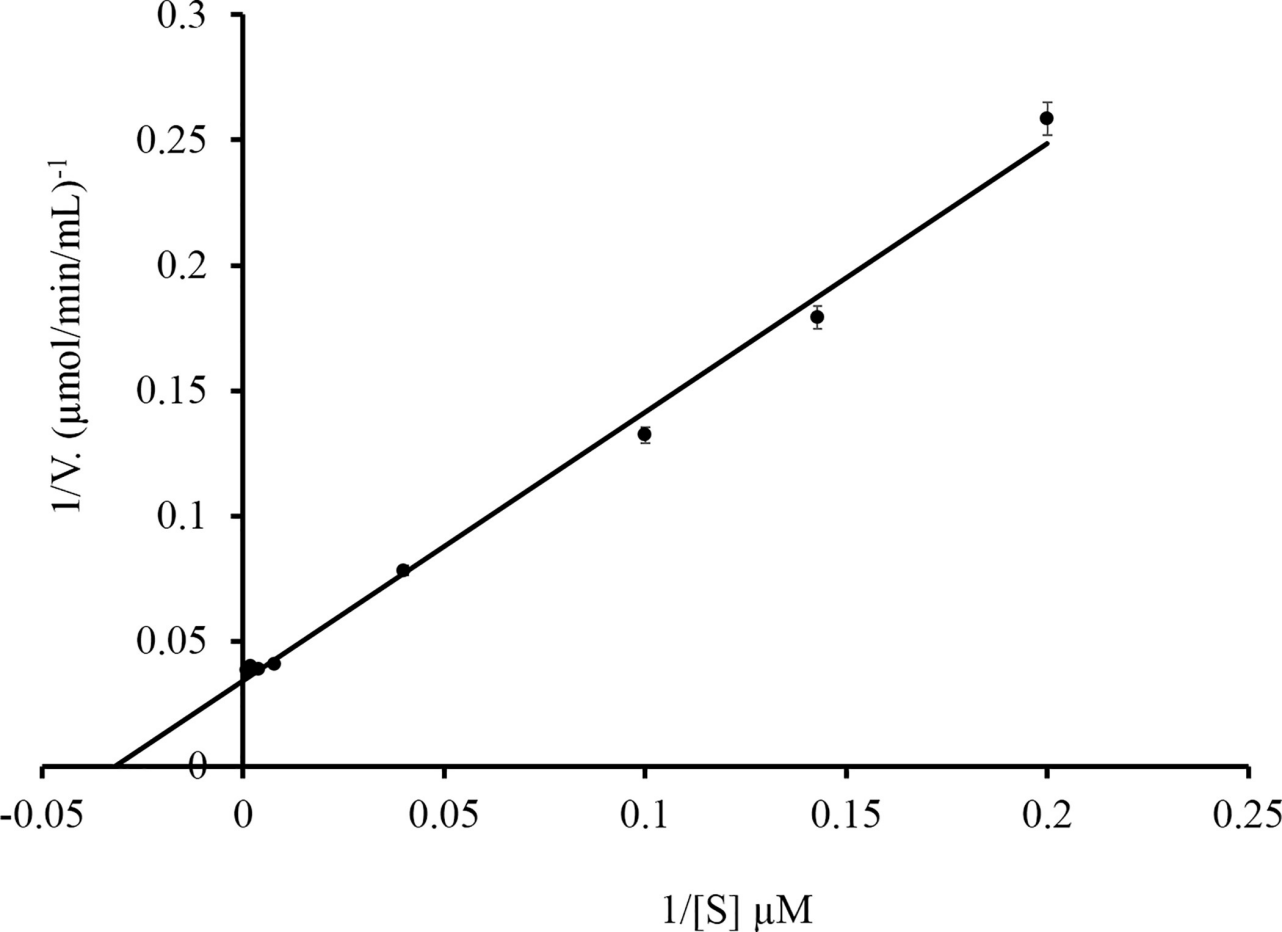
Lineweaver-Burk plot for recombinant cholesterol oxidase in the presence of various concentrations of cholesterol (0–1 mM).

The activity of the recombinant ChO was determined at different temperatures and pH. The optimum temperature for ChO activity was determined to be 70°C (Fig 7A). Nevertheless, the enzyme retained more than 60% of its activity at the temperatures between 50°C to 80°C under the tested conditions. Under various pH conditions, maximal activity of the enzyme was observed at pH 6 (Fig 7B). To calculate the K_m_ and V_max_ values of purified ChO, the activity of the enzyme was assayed with a range of cholesterol concentrations (0-1mM) at 25°C, 0.1 M potassium phosphate buffer pH 7. For K_m_ and V_max_ estimation, 1/V was plotted against 1/[S] in a Lineweaver-Burk plot, with K_m_ and V_max_ values obtained as 31 μM and 29.23 μmol/min/ mL (Fig 8).

## Discussion

Cholesterol oxidase, as an efficient biotechnological tool, is widely used across various industrial and clinical fields [30]. This enzyme is produced from a wide range of bacterial microorganisms with different enzymatic characteristics [5]. Since the thermal stability of the industrial and clinical enzymes is of great importance, selection of an enzyme with a high thermal stability and its successful over-expression could be very valuable. In the current study, we optimized the production of cholesterol oxidase from *Chromobacterium* sp. DS1 which is more stable at high temperatures than other commercially available ones.

Selecting the suitable *E*. *coli* host strain is an essential step for the overexpression of a recombinant protein. This can be achieved by considering the expression plasmid in combination with the nature of heterologous protein. For this purpose, *ChO*-encoding gene was expressed in various *E*. *coli* hosts using the pET expression system. *Rosetta-gami*2*(DE*3*)* produced high levels of active enzyme in comparison to the other host strains. pET expression system based on T7 promoter was used for efficient expression of our desired gene. The *DE*3 strains of *E*. *coli* hosts are preferred for expression of recombinant proteins in plasmids containing T7 promoters. The reason is that this strains contain a copy of *T7 polymerase* gene on their chromosome which produces RNA polymerase for this promoter [31]. On the other hand, the nature of the desired gene has a significant effect on the yield of recombinant protein in different hosts. On the other hand, one of the most common problems encountered in heterologous protein expression is rare codons. To address this problem, two strategies are utilized [32]: one approach is replacing the rare codons with common ones in a new synthetic gene. This strategy in some cases can lead to decreased mRNA stability and protein yield [33]. The second approach is the expression of heterologous protein in engineered strains containing rare tRNAs [34]. An example of such engineered strains is the E. coli Rosetta strain which carries pRARE plasmid providing tRNA genes for the rare codons.

Furthermore, the culture medium composition affects the yield of the recombinant protein expression by altering the bacterial host metabolism [35]. Therefore, culture medium should be accurately selected to achieve the maximum possible value of recombinant protein. In this regard, the effect of three different media (LB, SB, and TB) in the production of the recombinant ChO was evaluated. The results indicated that TB was the best option for the production of our desired protein. Superior buffering capacity, high concentration of yeast extract, and the use of glycerol as the carbon source supplement enable high biomass accumulation and high ChO production [36].

The appropriate bacterial growth phase at the time of expression induction and the optimal inducer concentration for maximal ChO production were determined individually. The results indicated that the yield of the enzyme was maximized when induction was made in the mid-exponential phase. On the other hand, evaluation of the biomass production under induction at different growth phases revealed that while the IPTG was added in the early exponential phase, biomass accumulation was lower than that of the stationary growth phase. Indeed, biomass production gradually rose when induction was made at the higher OD600. When induction was made in the early exponential growth phase, bacterial metabolic resources were channeled to produce recombinant protein comprising up to 50% of the total cellular protein [37, 38]. Based on this reasoning, low cellular growth rate would be expected following early exponential phase induction. It was also observed that 0.1 mM IPTG concentration was large enough to induce ChO protein expression.

To investigate the effect of different post-induction temperatures and the post-induction incubation time on the yield of recombinant ChO, three incubation temperatures (25°C, 30°C, and 37°C) were evaluated at different incubation times (6, 8, 16, and 24 h). Several studies have suggested that post-induction temperature as well as incubation time can affect the yield of recombinant protein production [39–42]. In addition, it was reported that different expression temperatures led to different enzymatic activities, although equal amounts of enzymes were synthesized at each temperature [43]. According to our results, reduction of the temperature down to 30°C along with incubation time of 16 h enhanced the enzyme yield by approximately three times relative to the same condition at 37°C. Generally, metabolic burden is usually observed in bacteria-producing heterologous proteins [44]. High-rate produced recombinant proteins may accumulate in insoluble aggregates (inclusion body) as a direct consequence of overwhelming the host folding machinery [45]. In addition, hydrophobic interactions which constitute a key factor in the formation of inclusion bodies diminish if temperature is reduced [46–48].

In the current study, optimization of the recombinant ChO has resulted in 22.98-fold increase (2.115 U/mL) in comparison with the un-optimized condition (0.092 U/mL). El-Naggar et al. optimized cholesterol oxidase production from two *Streptomyces* strains in their original producer microorganisms. They reported an overall 4.97-fold increase (15.631 U/mL) in *Streptomyces aegyptia* COX production as compared with the un-optimized condition (3.1 U/mL) [49]. In the second study, El-Naggar et al. found that the COX production by *Streptomyces cavourensis* after optimization process was 20.521 which is 6.19-fold higher than result obtained from un-optimized condition (3.31 U/mL) [50].

One step purification procedure was performed for recombinant ChO purification. In the end, the enzyme was purified 3.1 fold and overall yield of 82.5% was achieved. Dokyou et al. performed two step purification procedure for ChO isolation from *E*.*coli* Rosetta [51] and *Chromobacterium* sp.DS1 [52]. They used DEAE-cellulose DE52 column and achieved overall yield of 13% and 40% from *E*.*coli* Rosetta and *Chromobacterium* sp.DS1 respectively.

Characterization of the purified recombinant ChO indicated that the optimum temperature for the enzyme activity was 70°C at pH 7 and the enzyme retained more than 60% of its activity between 50°-80°C. In comparison, optimum temperature for *Brevibacterium sterolicum* and *Burkholderia cepacia* cholesterol oxidase were reported to be 50°C and 60°C respectively [51]. Our result is in agreement with the result of Doukyu et al. (2009), suggesting that the cholesterol oxidase from *Chromobacterium* sp. DS1 is an active enzyme across a wide range of temperatures. Moreover, such a high optimum temperature (70ºC) have been reported for COXs from *Pseudomonas aeruginosa* [53] and *Streptomyces fradiae* [51]. Furthermore, activity assay at different pH revealed that the optimum pH for enzyme activity was 6. Various studies reported optimum pH of 6 for different cholesterol oxidases for example the COX from *Brevibacterium sterolicum* [54], *Rhodococcus* sp. [3], and *Streptomyces* sp. [12]. The K_m_ value for the cholesterol was calculated to be 31 μM. This value is 5 μM larger than the value calculated by Doukyu et al. for cholesterol oxidase from *Chromobacterium* sp. DS1. On the other hand, the calculated K_m_ was lower than that of enzymes from *Streptomyces* sp. SA-COO, *B*. *cepacia* ST-200, and *P*. *fluorescens*, but not that from *Nocardia* species [27].

In conclusion, our study indicated that the yield of recombinant ChO was markedly increased when ChO was produced under optimized protocol. In addition, purification and characterization of recombinant ChO verified again that cholesterol oxidase from *Chromobacterium* sp. DS1 is a thermo-stable enzyme with a broad range of thermal activity.
